# Controlled synthesis of conjugated polycarbazole polymers via structure tuning for gas storage and separation applications

**DOI:** 10.1038/s41598-017-10372-4

**Published:** 2017-11-13

**Authors:** Guoyan Li, Long Qin, Chan Yao, Yanhong Xu

**Affiliations:** 1 Key Laboratory of Preparation and Applications of Environmental Friendly Materials of the Ministry of Education, Jilin Normal University, Changchun, 130103 China; 2 Key Laboratory of Functional Materials Physics and Chemistry of the Ministry of Education, Jilin Normal University, Siping, 136000 China

## Abstract

A series of conjugated microporous polymers (CMPs) based on 1,3,6,8-tetrabromocarbazole (N_4_CMP-1–5) is synthesized via Suzuki cross-coupling or Sonogashira polycondensation. The porosity properties and surface area of these polymer networks can be finely tuned by using a linker with different geometries or strut length. These polymers show the Brunauer-Emmett-Tellerthe (BET) surface areas ranging from 592 to 1426 m^2^ g^−1^. The dominant pore sizes of the polymers on the basis of the different linker are located between 0.36 and 0.61 nm. Gas uptake increases with BET surface area and micropore volume, N_4_CMP-3 polymer can capture CO_2_ with a capacity of 3.62 mmol g^−1^ (1.05 bar and 273 K) among the obtained polymers. All of the polymers show high isosteric heats of CO_2_ adsorption (25.5–35.1 kJ mol^−1^), and from single component adsorption isotherms, IAST-derived ideal CO_2_/N_2_ (28.7–53.8), CO_2_/CH_4_ (4.6–5.2) and CH_4_/N_2_ (5.7–10.5) selectivity. Furthermore, N_4_CMPs exhibit the high CO_2_ adsorption capacity of 542–800 mg g^−1^ at 318 K and 50 bar pressure. These data indicate that these materials are a promising potential for clean energy application and environmental field.

## Introduction

In recent years, the global climate change mainly caused by excessive carbon dioxide (CO_2_) emissions has drawn great attentions and concerns^1^. Developing viable CO_2_ capture and storage (CCS) technologies to stabilize atmospheric CO_2_ levels and cope with global warming, which is an effective way^2^. Porous organic polymers (POPs) constructed by low mass density, non-metallic elements, not only have a large specific surface area, high pore volume, narrow pore size distribution, good chemical and physical stability, and wide synthetic diversification, but also present cost and effective gas uptake applications^3–5^. POPs physisorb CO_2_ molecules via weak van der Waals forces, which are potential candidates for CO_2_ capture because of their low regeneration energy consumption and high CO_2_ sorption capacity^6^. In the past few years, versatile POPs materials such as covalent organic frameworks (COFs)^7,8^, covalent triazine-based frameworks (CTFs)^6^, polymers of intrinsic microporosity (PIMs)^9,10^, porous aromatic frameworks (PAFs)^11^, conjugated microporous polymers (CMPs)^12^, and hypercross-linked polymers (HCPs)^13^, have been rapidly developed due to their important applications in a broad variety of aspects including gas storage/separations^14,15^, chemosensors^16–18^, tunable photoluminescence^19,20^, heterogeneous catalysis^21,22^ and so on.

Conjugated microporous polymers (CMPs) are a new class of porous materials, which are synthesized by transition metal coupling chemistry including Pd-catalyzed Suzuki and Sonogashira cross-coupling polycondensation^23^, Ni-catalyzed Yamamoto reaction^24^, and other reactions such as oxidative polymerization^25^, Schiff-base reaction^26^. The unmatched feature of CMPs is that they combine π-conjugation and permanent porous structure in a bulk material. Recently, some reports have revealed that the introduction of some polar functional groups or heteroatoms into porous materials could enhance the binding affinity between the adsorbent and CO_2_ molecules, which leads to the increase of CO_2_ capture capacity^27–30^. In this work, we chose carbazole as the network core and different flexible monomers as linker space to construct the final network based on the following reasons: (1) carbazole-based porous organic polymers have been studied as strong candidates for carbon dioxide (CO_2_) due to the rigid structure and special intrinsic properties of their building blocks; (2) carbazole possesses polar group (-NH) from carbazole unit, which might promote the interaction between the solid adsorbent and acidic CO_2_ molecules; (3) conjugated property of rigid carbazole unit is beneficial for formation of porous structure with permanent porosity and stability; (4) the linker monomers possess different steric configuration, such as linear type, triangle, and tetrahedral, which can exhibit different flexibility to form the microporous volume. Therefore, introduction of the carbazole unit into the polymer skeleton makes the porous materials electron-rich to enhance CO_2_ uptake of porous polymers, and the steric configuration of linker monomer can effectively construct and adjust the microporous volume and reach outstanding gas storage and separation capacity^31–33^.

**Figure 1:**
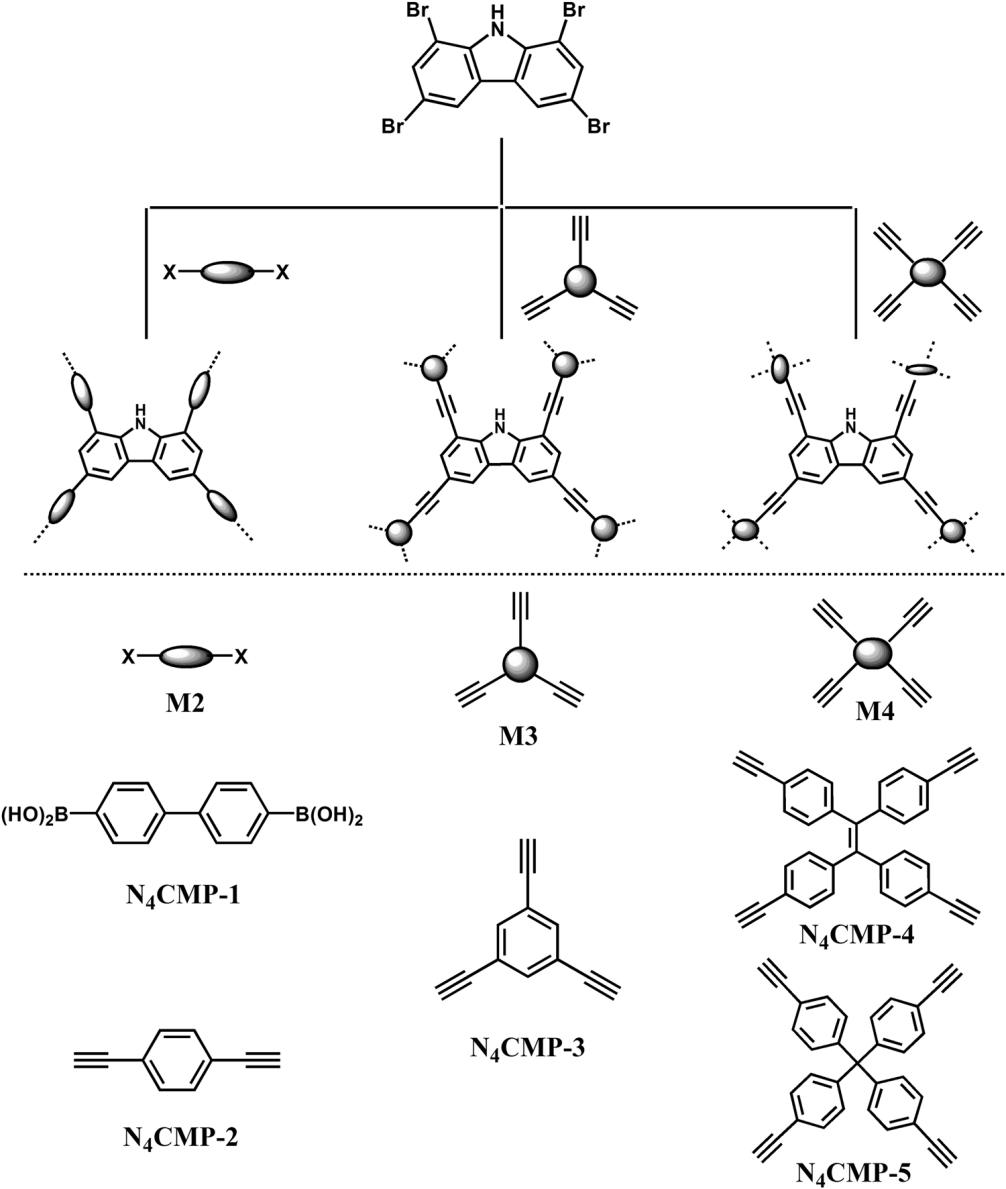
Schematic representation of synthesis of N_4_CMP polymers.

With these considerations in mind, herein, we prepared a series of CMPs (Fig. 1, N_4_CMP-1–5) based on 1,3,6,8-tetrabromocarbazole as the basic buliding block via Pd-catalyzed Suzuki cross-coupling or Sonogashira polycondensation. The porosity properties and surface area in the kind of amorphous CMPs can be finely controlled by the strut length, size or geometry of linker. The obtained carbazole-based CMPs possess large specific surface area, high micropore volume, narrow pore size distribution, high gas uptake capacity, and good selectivity toward CO_2_ over N_2_ or CH_4_. Meanwhile, N_4_CMP polymer networks also exhibit high CO_2_ adsorption capacity at high pressure condition.

## Results

### Synthesis and characterization

All of the polymer networks were synthesized by palladium (0)-catalyzed cross-coupling polycondensation of 1,3,6,8-tetrabromocarbazole and a number of benzeneboronic monomers or ethynyl monomers. All the reactions were carried out at a fixed reaction temperature and reaction time (120 °C/48 h). The general synthetic route towards N_4_CMPs polymers is shown in Scheme 1. Our aim is to explore the effect of structure and connecting position of linker on pore properties of the resulting porous polymers. The insoluble polymers were filtered and washed with water, tetrahydrofunan, chloroform and methanol, respectively, in order to remove the inorganic salts, organic monomers, residual catalyst, and oligomers. All of these polymers are insoluble in common organic solvents because of their highly cross linked structures.

**Figure 2:**
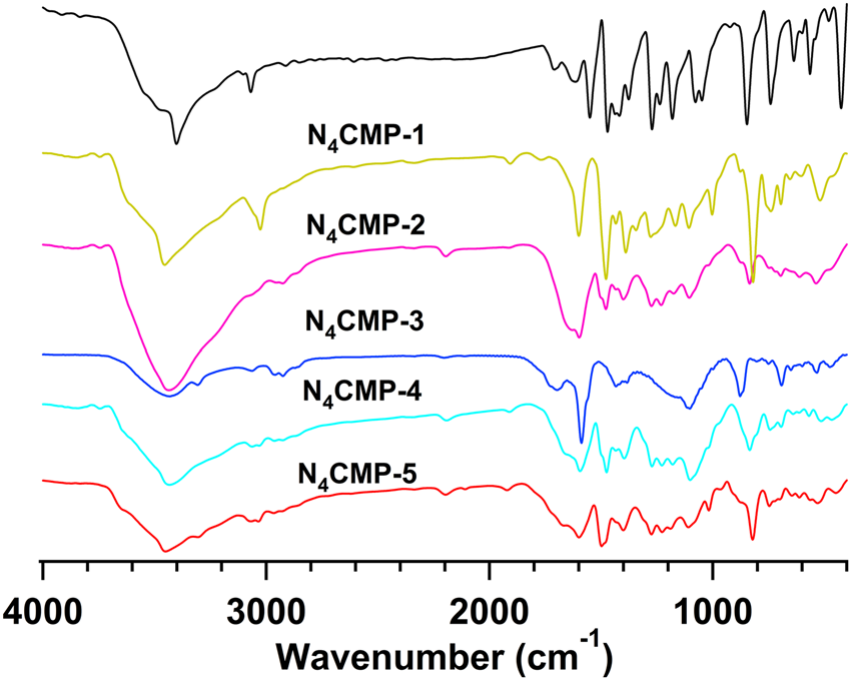
FT-IR spectra of 1,3,6,8-tetrabromocarbazole (black), and polymers N_4_CMP-1–5.

The structures of N_4_CMPs polymers were further characterized by Fourier transform Infrared (FT-IR) spectroscopy (Fig. 2). All the polymer networks show the characteristic C=C stretching band at 1600 cm^−1^ and aromatic C–H stretching frequencies up to 3000 cm^−1^. All the spectra exhibit intense characteristic bands of N-H at about 3450 and 1390 cm^−1^ ^31–33^. The band around 1500 cm^−1^ is assigned to the stretching vibration of C-N-C in the five-membered NC_4_ ring for all the samples^31–33^. The primary bromo group of 1,3,6,8-tetrabromocarbazole at about 590 cm^−1^ are absent in the polymer networks. The typical C≡C stretching mode at about 2200 cm^−1^ is also observed in the N_4_CMP-2, N_4_CMP-3, N_4_CMP-4, and N_4_CMP-5, respectively. These results suggested that a hyper-cross-linked structure was successfully obtained. The structures of N_4_CMPs polymers were characterized at the molecular level by solid state^13^C cross-polarization magic-angle spinning (CP/MAS) NMR (ESI, Figure S1). In general, N_4_CMP-1–5 have the similar broad peaks. The resolved resonance peak at about 137 ppm arises from the carbons of the carbazole rings. Two peaks at about 130, 123, and 110 ppm can be ascribed to the other carbons in the aromatic rings. The small peaks at about 93 ppm correspond to acetylene carbons from acetylene monomers. These peaks are perfectly consistent with previous works^31–33^.

**Figure 3:**
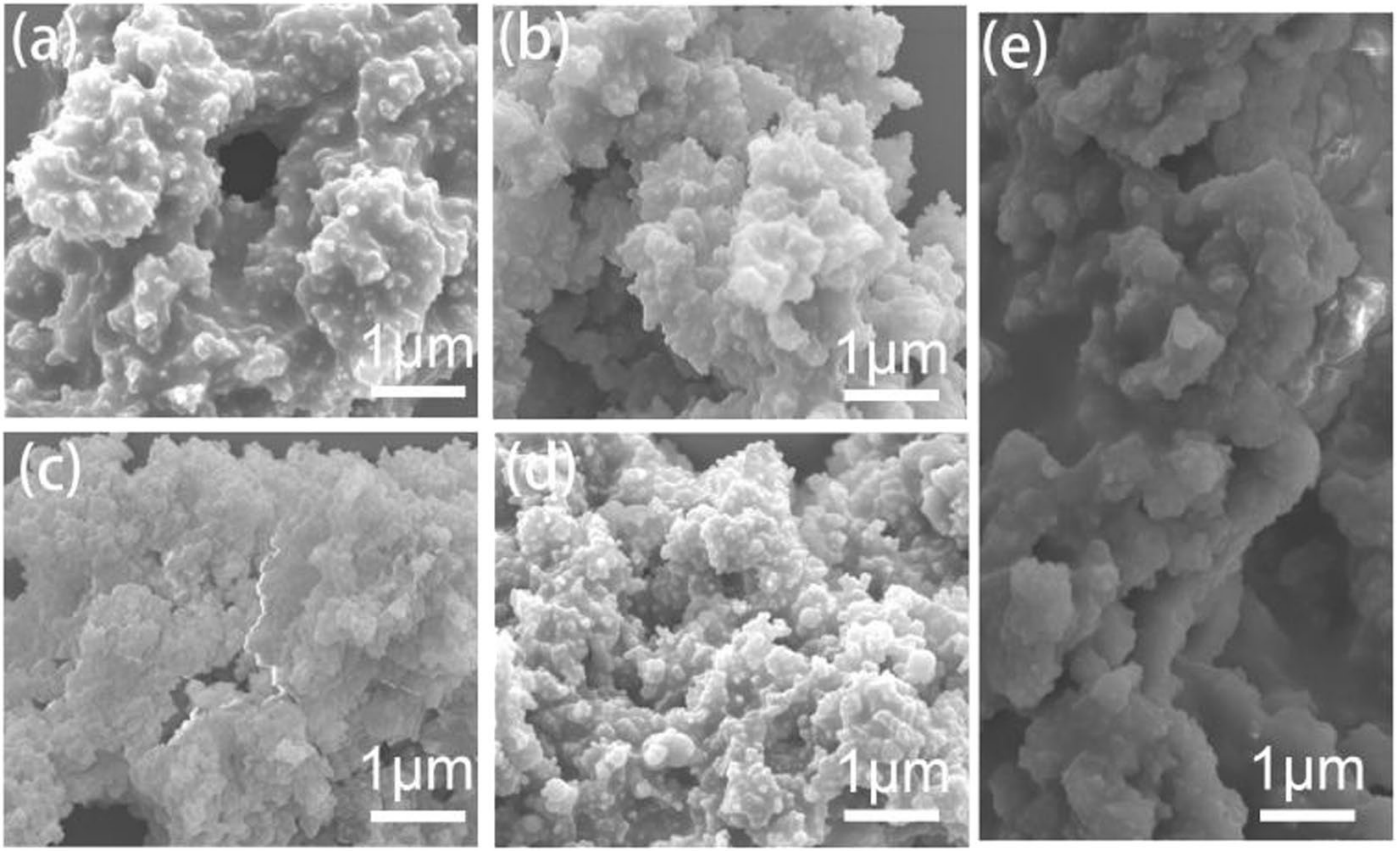
FE-SEM images of (**a**) N_4_CMP-1, (**b**) N_4_CMP-2, (**c**) N_4_CMP-3, (**d**) N_4_CMP-4, and (**e**) N_4_CMP-5.

The morphology information was collected by field-emission scanning electron microscopy (FE-SEM) and high-resolution transmission electron microscopy (HR-TEM). The typical SEM images of N_4_CMPs indicate that all the polymers have irregular shapes with sizes of about several micrometers (Fig. 3). The TEM images revealed that they are porous structures of the materials, which is similar to some reported amorphous microporous organic materials (ESI, Figure S2)^34–36^. The UV/vis diffuse reflectance spectra of the N_4_CMP are displayed in Figure S3. Both polymers showed a broad absorption range. All the five polymers displayed a broad absorption with wavelengths ranging from 375 to 428 nm. Although the shapes of the spectra are similar, they do display differences in terms of the onset of the absorption, which correlates with the band gap of the conjugated system. The TGA results verify that the polymers have a good thermal stability, and the thermal degradation temperature is up to ca. 360 °C (ESI, Figure S4). The weight loss below 100 °C is generally attributed to the evaporation of adsorbed water and gas molecules trapped in the micropores. Powder X-ray diffraction (PXRD) measurements indicated that all the polymers are amorphous in nature (ESI, Figure S5), as most other reported CMP networks^12^.

**Figure 4:**
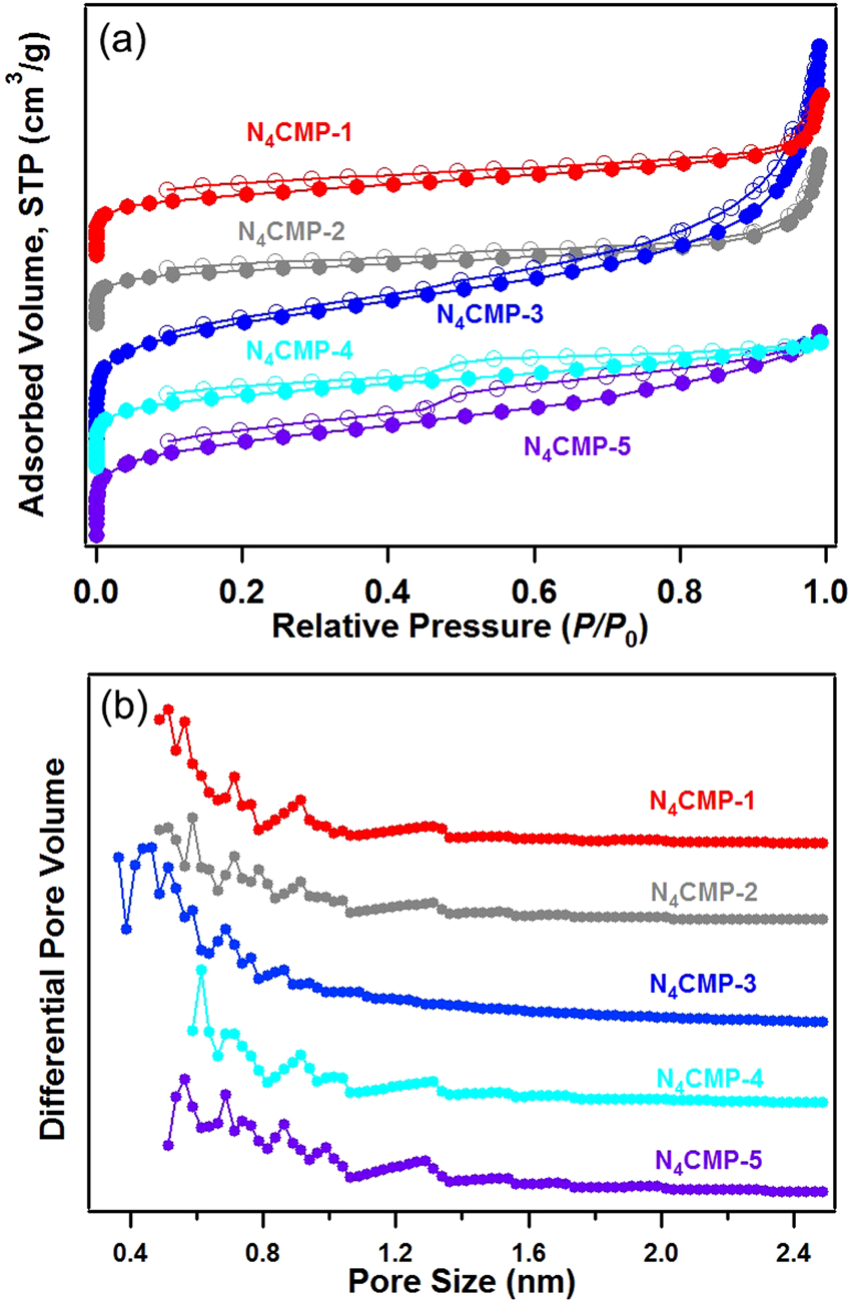
(**a**) Nitrogen sorption isotherms measured at 77 K for N_4_CMP-1–5, the isotherms of N_4_CMP-1–4 are shifted vertically by 800, 600, 400 and 200 cm^3^ g^−1^ for better visibility, respectively; (**b**) pore size distributions calculated using nonlocal density functional theory (NLDFT) method, for clarity, the curves of N_4_CMP-1–4 are shifted vertically by 4, 3, 2 and 1 cm^3^ g^−1^, respectively.

The porosity of the polymer networks was investigated by nitrogen adsorption/desorption experiments at 77 K, in which the fully reversible isotherms exhibit rapid nitrogen uptake at low relative pressures (*P/P*_0_ < 0.01), indicates that the five polymers belong to microporous materials (Fig. 4a and Figure S6). Meanwhile, all the polymer networks gave rise to type I nitrogen gas sorption isotherms according to the IUPAC classification^21^. N_4_CMP-1, N_4_CMP-2 and N_4_CMP-3 show little hysteresis upon desorption, suggesting that adsorption and desorption are almost equally facile. Nevertheless, N_4_CMP-4 and N_4_CMP-5 networks show an evident hysteresis loop, which is partly attributed to the swelling in a flexible polymer network, as well as mesopore contribution^34,35^. In fact, the described low-pressure hysteresis is common phenomenon in a variety of microporous polymer networks^18,19,36–41^. The presence and magnitude of this hysteresis may be an indication of the softness of the material. Besides, a sharp rise in the high pressure region (*P/P*_0_ > 0.8) is also observed in the sorption isotherms of N_4_CMP-1, N_4_CMP-2 and N_4_CMP-3, respectively, suggesting that the materials possess some macropores, which is attributed to the nitrogen condensation in interparticular voids formed by the aggregation of polymer microspheres observed in the SEM images, which is similar to the previous reports^42,43^. The apparent BET surface areas for the networks (Table S1) were calculated over a relative pressure range *P*/*P*_0_ = 0.015–0.1, which was found to give a positive value of C in the BET equation. Polymer N_4_CMP-3 shows the highest BET surface areas with a value of 1426 m^2^ g^−1^, while N_4_CMP-2 represents the lowest value of 592 m^2^ g^−1^ among the five polymer networks. As a general trend, when the similar synthetic methodology was used, polymers prepared from longer linkers showed lower surface areas than those from shorter linkers, the similar results were reported by the Cooper and Thomas group^23,24,36^. For example, N_4_CMP-1 had a BET surface area of 650 m^2^ g^−1^, which decreased to 592 m^2^ g^−1^ for N_4_CMP-2. This phenomenon is similar to that of reported previously, in which CMPs constructed with longer connecting struts have lower BET surface areas^23,36^. Beyond this, Jiang *et al*. reported the linkage geometry between the connecting nodes could affect pore properties, conjugation and surface area for CMP networks^37^. Their experiment results demonstrated that the *meta*-linkage is superior to the *ortho*- and *para*-linkages for the construction of porous skeletons. This phenomenon is also observed in this system, for example, polymer N_4_CMP-3 containing the *meta*-linkage 1,3,5-triethylbenzene possesses the highest surface area (1426 m^2^ g^−1^), which is higher than that of polymers N_4_CMP-4 (995 m^2^ g^−1^) and N_4_CMP-5 (1347 m^2^ g^−1^) with the *ortho*- and *para*-linkage. In addition, the polymer N_4_CMP-5 shows a higher surface area in the contrastion of N_4_CMP-4, possible reason is that the effect of steric hindrance from tetrakis(4-bromophenyl)methane improved the further growth of the molecular weight of the polymer during polymerization, which leads to the increase of surface area. The micropore volume was calculated from the adsoprtion branch of the nitrogen adsorption-desorption isotherm using the *t*-plot method (ESI, Figure S7). A summary of the BET specific surface area and porous properties is shown in Table S1.

The pore size distribution (PSD) of polymers N_4_CMPs was calculated from the related adsorption branch of the isotherms by the NLDFT method. As shown in Fig. 4b, the N_4_CMPs polymers based on different building blocks showed the dominant pore sizes range between 0.36 and 2.0 nm. Polymers N_4_CMP-1 and 2 possessing same core structures were synthesized by bis-substituted monomers with the increasing length (biphenylene, diethynylbenzene), and N_4_CMP-3–5 were prepared with different geometry linkage nodes. For instance, N_4_CMP-3 has smaller micropore diameter (0.36 nm) and higher surface area compared to other four polymers, this result could be explained by the fact that the *meta*-linkage is superior to the *ortho*- and *para*-linkages for the construction of porous skeletons, similar results were observed for other reported CMPs^24,31,37^. Meanwhile, from the N_2_ sorption isotherm, the ratio of micropore volume to total pore volume (*V*_Micro_/*V*_Total_) can be calculated which describes the degree of microposity. All the five polymers exhibited the *V*_Micro_/*V*_Total_ values above 0.55, ranging from 0.57 to 0.79, which indicates that a high fraction and dominance of micropores in these networks. These results implyed that the BET surface area and porosity porosities in the CMP networks could be finely controlled by using either linker with different strut length or different geometries. The key structural properties of polymers derived from the corresponding isotherm are listed in Table S1, such as the BET specific surface area, micropore surface area, pore volume, micropore volume, dominant pore size, gas uptake, and selectivity.

## Discussion

### Gas uptake capacity and separation

**Figure 5:**
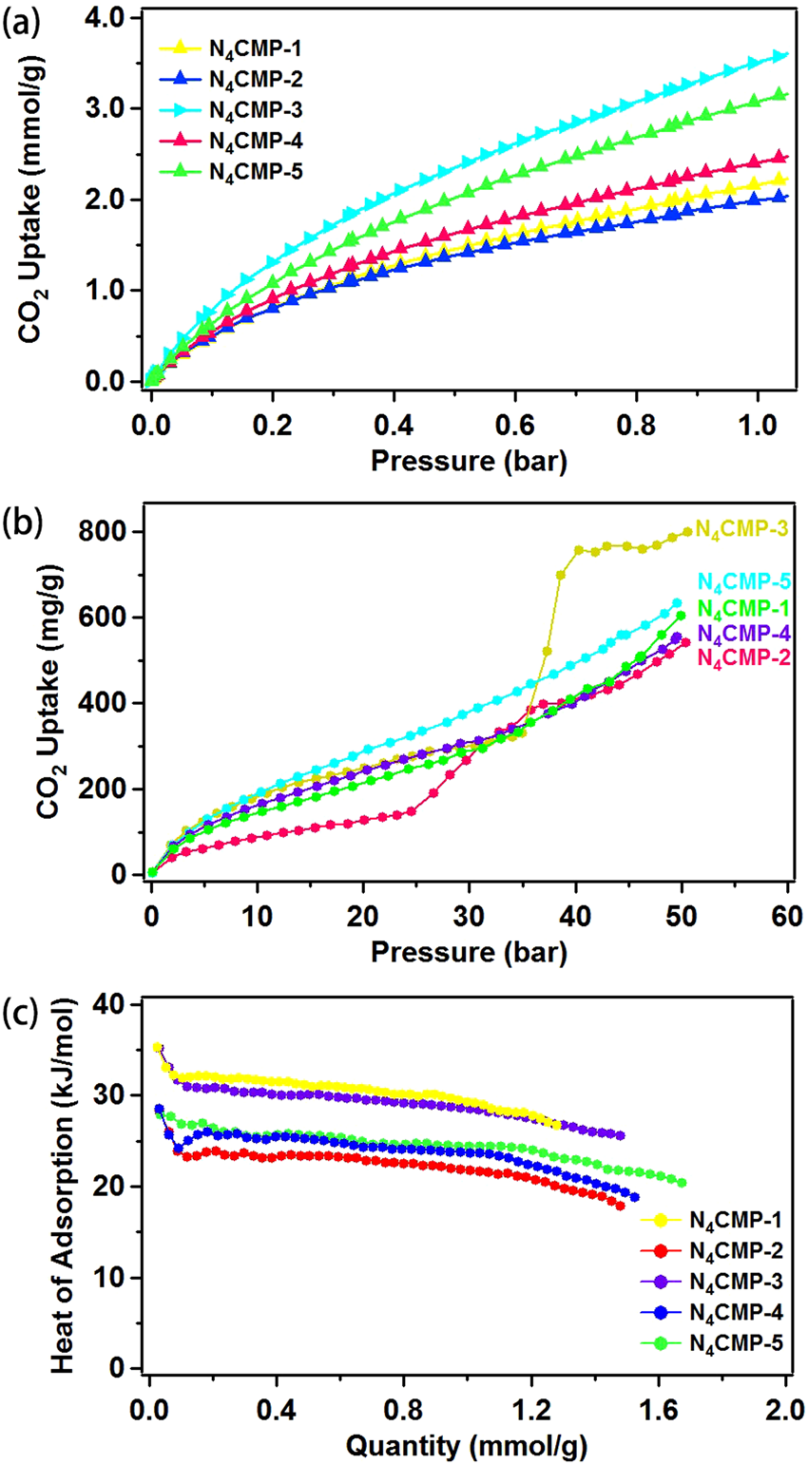
CO_2_ adsorption isotherms collected (**a**) at 273 K and 1.05 bar and (**b**) at 318 K and 50 bar, respectively; (**c**) The isosteric heat of adsorption for N_4_CMPs.

It has been proved that compare to other most porous polymers, carbazole-based polymer networks can efficiently adsorb gas (hydrogen and carbon dioxide) under the same conditions^32,38–41^. Therefore, gas uptake capacities of the nitrogen-rich polymers were investigated. The CO_2_ uptakes of the polymer networks were studied at 1.05 bar and different temperature (273 K and 298 K) (Fig. 5a and Figure S8). We find that an increasing trend in the carbon dioxide loading capacity (from 2.05 mmol g^−1^ to 3.62 mmol g^−1^) with increasing micropore surface area and pore volume (Table S1). N_4_CMP-3 with the highest micropore surface area and micropore volume, shows the highest CO_2_ uptake of 3.62 mmol g^−1^ at 273 K and 1.05 bar among the resulting polymer networks, followed by N_4_CMP-5 (3.18 mmol g^−1^), N_4_CMP-4 (2.49 mmol g^−1^), N_4_CMP-1 (2.24 mmol g^−1^), and N_4_CMP-2 (2.05 mmol g^−1^) (Table S2 in the Supporting Information). From this result, we found that N_4_CMP-1 and N_4_CMP-2 have similar micropore surface area, however, N_4_CMP-1 shows the relatively higher CO_2_ uptake, possible reason is that N_4_CMP-1 has high micropore volume (Table S1). Interestingly, these CO_2_ uptake values are not only significantly higher than many microporous materials with a similar specific surface area but also comparable to the reported large surface area of porous aromatic frameworks under the same conditions, such as PAF-1 (2.1 mmol g^−1^, *S*_BET_ = 5640 m^2^ g^−1^)^44^, COF-102 (1.56 mmol g^−1^, *S*_BET_ = 3620 m^2^ g^−1^)^45^, and PP-CMP@mmm (2.52 mmol g^−1^, *S*_BET_ = 1928 m^2^ g^−1^)^37^, but lower than TSP-2 (4.1 mmol g^−1^, *S*_BET_ = 913 m^2^ g^−1^)^40^, ALP-1 (5.4 mmol g^−1^, *S*_BET_ = 1235 m^2^ g^−1^)^46^ and PPF-1 (6.1 mmol g^−1^, *S*_BET_ = 1740 m^2^ g^−1^)^47^, implying that the surface area of porous polymers is not a sole factor in determining the capacity of CO_2_ uptake. The superior adsorption properties of the N_4_CMP polymers can be ascribed to the enhanced dipole-quadrupole interactions and/or the strong interactions of the polarizable CO_2_ molecules through hydrogen bonding (from the N-H group), similar results were also observed for previously reported carbazole-based CMPs^23^. The recycling is an important parameter for their practical application. We tested the reusability of N_4_CMP-3, N_4_CMP-4 and N_4_CMP-5 at 1.05 bar and 273 K, and found the samples could be efficiently recycled and reused for four cycles without significant loss of CO_2_ uptake (ESI, Figure S9).

Furthermore, a high pressure CO_2_ sorption properties of the polymers were also investigated at 50 bar and 318 K. As seen in Fig. 5b, N_4_CMP-2 and N_4_CMP-3 produce a type IV isotherm according to IUPAC classifications^48^. N_4_CMP-1, N_4_CMP-4 and N_4_CMP-5 show a nearly linear increase with the increasing pressure no obviously turning point. All the polymers show similar growth trend in the capture of carbon dioxide gas (from 542 mg g^−1^ to 800 mg g^−1^) with increasing surface area and pore volume under high pressure condition. N_4_CMP-3 exhibits the highest CO_2_ capture capacity of 800 mg g^−1^. N_4_CMP-1, N_4_CMP-2, N_4_CMP-4, and N_4_CMP-5, exhibit the CO_2_ uptake of 605, 542, 556, and 695 mg g^−1^, respectively. These results indicate that electron-rich polymer backbone, porosity property and pressure play a positive role in the increase of gas capature capacity.

To further understand the pore surface properties and the adsorption process, the isosteric heat (*Q*_*s*t_) of polymers N_4_CMP for adsorption CO_2_ is calculated based on adsorption isotherms of CO_2_ at 273 K and 298 K in terms of the Clausius–Clapeyron equation (Fig. 5c)^49^. The *Q*_st_ values of CO_2_ drop with loading amount, meaning that the interaction between CO_2_ and the pore wall is stronger than that between CO_2_ molecules. The *Q*_*s*t_ values of CO_2_ adsorption for all the polymer networks range from 25.5 to 35.1 kJ mol^−1^ at the near zero coverage, which can be comparable to other carbazole-based polymers and higher than those of some reported POPs, such as polycarbazole CPOP-1 (24.5–30.2 kJ mol^−1^)^38,40,41^, HCP materials (20–24 kJ mol^−1^)^50^, polybenzimidazole BILP polymers (26.7–28.8 kJ mol^−1^)^51^. The higher *Q*_st_ value may be attributed to the appropriate pore structures for CO_2_ adsorption, electron-rich polycarbazole network and high charge density at the nitrogen sites of polymers that can enhance the interaction between CO_2_ molecules and the pore surfaces of N_4_CMP.

**Figure 6:**
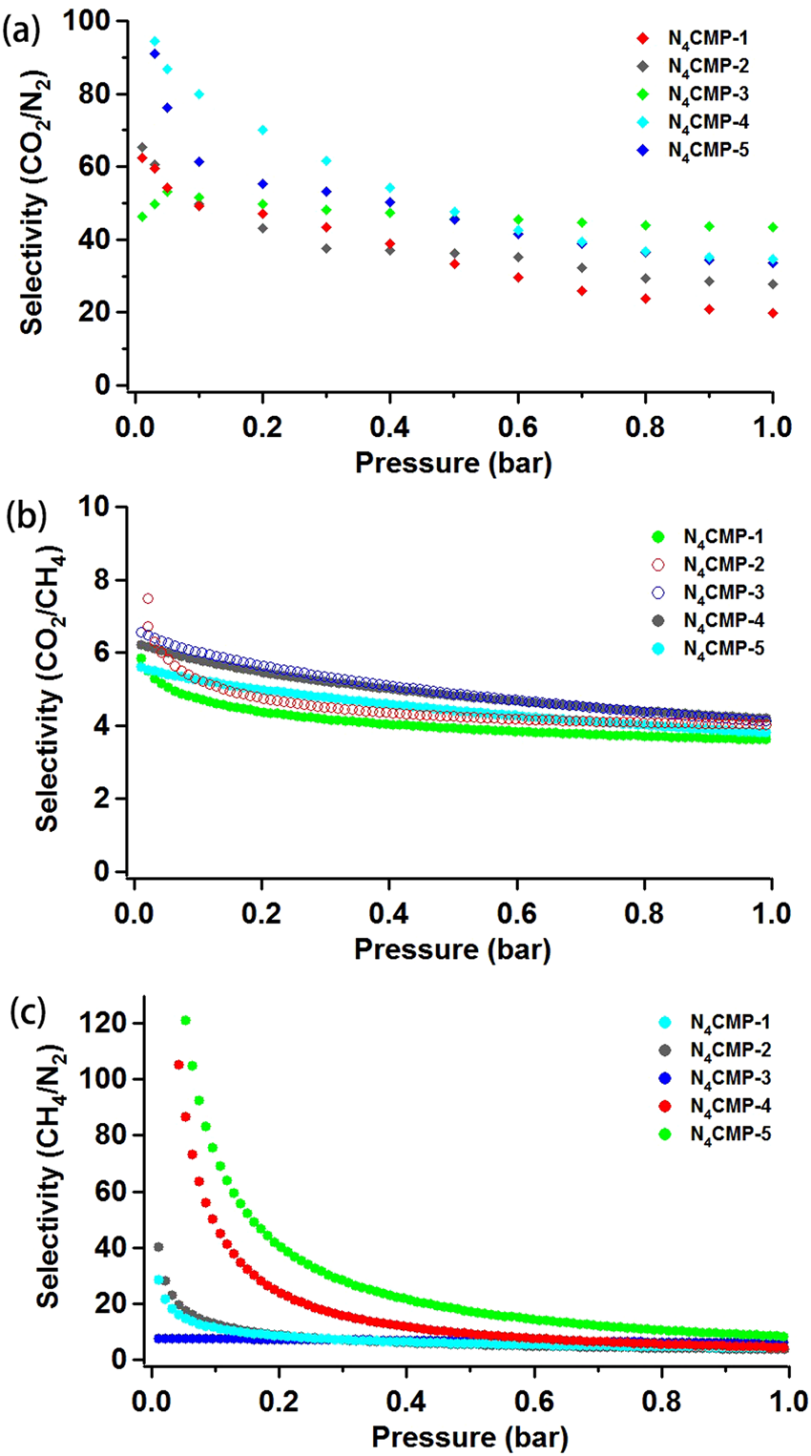
IAST selectivity for 0.15/0.85 CO_2_/N_2_ mixture, 0.50/0.50 CO_2_/CH_4_ mixture, and 0.50/0.50 CH_4_/N_2_ mixture.

Besides gas storage, the selectivity is very important for potential application in gas separation. Considering the good gas adsorption performance of the obtained polymers, the gas selective adsorption behaviors of N_4_CMP polymers are measured. The sorption experiments of CO_2_, CH_4_, and N_2_ were carried out at 273 K and 1.05 bar, respectively. The CO_2_ or CH_4_ uptake shows a almost linear increase with the increasing pressure, whereas that of nitrogen has no apparent increase trend (ESI, Figure S10). From the available single-gas adsorption isotherms, the selectivity in the adsorption of CO_2_, CH_4_ and N_2_ from CO_2_-N_2_, CO_2_-CH_4_, and CH_4_-N_2_ gas mixtures was estimated by Ideal Adsorption Solution Theory (IAST), which has been widely used to predict gas mixture adsorption behavior in the porous materials^52,53^. Under simulated flue gas streams (typically 15% CO_2_ and 85% N_2_), N_4_CMP-3 exhibits the highest selectivity for CO_2_/N_2_ among the five polymers (53.8 at 273 K and 1.05 bar) (Fig. 6a). This value is lower than that of porous benzimidazole linked polymers BILP 1–7 (59‒113)^54^ and PECONF 1‒4 (74‒109)^55^, while it is comparable to those of previously reported carbazole-based polymers (29.7‒35.4)^31^. In addition, the CO_2_/CH_4_ adsorption selectivity for N_4_CMP polymer networks is calculated to be 4.6–5.2 at 273 K and 1.05 bar, which is moderate value (Fig. 6b and Table S1). The CH_4_/N_2_ selectivity for N_4_CMP polymers is 5.7–10.5 (Fig. 6c and Table S1). Furthermore, we employ initial slopes ratio estimated from Henry’s law constants for single-component adsorption isotherms, which has been widely adopted by researchers^56^. the CO_2_ selectivities of N_4_CMP-1‒5 over N_2_ and CH_4_ were also calculated using initial slopes calculations at 273 K and 1.0 bar, the selectivities of CO_2_/N_2_ (46.2–69.1) (ESI, Figure S11, Table S1) and CO_2_/CH_4_ (6.4–12.1) (ESI, Figure S12, Table S1). The CH_4_/N_2_ adsorption selectivities range from 4.0 to 10.6 (ESI, Figure S13, Table S1). These excellent CO_2_ selective capture performances of N_4_CMP samples evaluated by Henry’s law are consistent with the results calculated from IAST. Due to their relatively high heats of adsorption and good selectivity of CO_2_/N_2_, CO_2_/CH_4_ and CH_4_/N_2_, the N_4_CMPs synthesized in this work could have potential for post-combustion CO_2_ capture.

## Conclusion

In conclusion, a series of carbazole-containing CMPs have been synthesized by palladium (0) catalyzed cross-coupling polycondensation. The study suggests that the monomer reactivity and the efficacy of chemistry have a pronounced effect on the surface areas and porosity attained in addition to the monomer geometry structure and linker strut length. All of the polymers are microporous with the BET surface areas ranging from 592 to 1426 m^2^ g^−1^ and possess quite narrow pore distributions at around 0.36 and 0.61 nm. The thermally stable polymer N_4_CMP-3 exhibits the highest CO_2_ adsorb uptake up to 3.62 mmol g^−1^ (1.05 bar/273 K) and 800 mg g^−1^ (50 bar/318 K) among the obtained polymers. All of the CMP networks show high isosteric heats of CO_2_ adsorption (25.5–35.1 kJ mol^−1^). We believe that this type of CMPs material would be a promising candidate for clean energy application and environmental-friendly field.

## Methods

### Synthesis of N_4_CMP-1

A mixture of 1,3,6,8-tetrabromocarbazole (100 mg, 0.21 mmol) and biphenyl-4,4′-diboronic acid (101.5 mg, 0.42 mmol) in dimethylformamide (DMF, 8 mL) was degassed by three freeze–pump–thaw cycles. To the mixture was added an aqueous solution of K_2_CO_3_ (1.0 M, 1 mL) and tetrakis(triphenylphosphine)palladium (0) (14.8 mg, 12.8 μmol), respectively. The resulting solution was further degassed for three cycles, and purged with N_2_, and stirred at 120 °C for 48 h. The mixture was cooled to room temperature, and then insoluble precipitate was filtered and washed with H_2_O, CH_3_OH, CHCl_3_, and THF to remove any unreacted monomers or catalyst residues. Further purification of the polymer was carried out by Soxhlet extraction with H_2_O, CHCl_3_, THF, and CH_3_OH for 24 h, respectively, **N**_**4**_ **CMP-1** (gray powder, 93 mg, 94.7% yield). Elemental Analysis (%) (**N**_**4**_ **CMP-1**) C 92.86, H 5.37, N 1.77; Found C 91.11, H 5.10, N 2.06.

### Synthesis of N_4_CMP-2, N_4_CMP-3, N_4_CMP-4, and N_4_CMP-5

1,3,6,8-Tetrabromocarbazole (100.0 mg, 0.21 mmol) and 1,4-diethynylbenzene (52.9 mg, 0.42 mmol) (**N**_**4**_ **CMP-2**)/1,3,5-triethynylbenzene (42 mg, 0.28 mmol) (**N**_**4**_ **CMP-3**)/1,1′,2,2′-tetrakis(4-ethynylphenyl)ethene (90 mg, 0.21 mmol) (**N**_**4**_ **CMP-4**)/tetrakis(4-ethynylphenyl)methane (87.5 mg, 0.21 mmol) (**N**_**4**_ **CMP-5**) were put into a 50 mL round-bottom flask, then the flask exchanged 3 cycles under vacuum/N_2_. Then added to 2 mL DMF and 2 mL triethylamine (Et_3_N), the flask was degassed by three freeze–pump–thaw cycles, purged with N_2_. When the solution had reached reaction temperature, a slurry of tetrakis(triphenylphosphine)palladium (0) (14.5 mg, 12.6 μmol) (**N**_**4**_ **CMP-2**)/(**N**_**4**_ **CMP-3**)/(**N**_**4**_ **CMP-4**)/(**N**_**4**_ **CMP-5**) in the 1 mL DMF and copper (I) iodide (3.8 mg, 0.02 mmol) (**N**_**4**_ **CMP-2**)/(**N**_**4**_ **CMP-3**)/(**N**_**4**_ **CMP-4**)/(**N**_**4**_ **CMP-5**) in the 1 mL Et_3_N were added, and the resulting solution was further degassed by freeze-pump-thaw for three cycles, and purged with N_2_, and stirred at 120 °C for 48 h, respectively. The mixture was cooled to room temperature, and then insoluble precipitate was filtered and washed with H_2_O, CH_3_OH, CHCl_3_, and THF to remove any unreacted monomers or catalyst residues. Further purification of the polymer was carried out by Soxhlet extraction with H_2_O, CHCl_3_, THF, and CH_3_OH for 24 h, respectively, to give **N**_**4**_ **CMP-2** (yellow solid, 81 mg, 94.5%), **N**_**4**_ **CMP-3** (brown solid, 71 mg, 95%), **N**_**4**_ **CMP-4** (black solid, 113 mg, 92%), **N**_**4**_ **CMP-5** (brownish black solid, 109 mg, 90.6%). Elemental Analysis (%) Calcd. (**N**_**4**_ **CMP-2**) C 94.24, H 3.65, N 2.11; Found C 92.88, H 3.87, N 2.44; (**N**_**4**_ **CMP-3**) C 94.97, H 3.19, N 1.84; Found C 92.78, H 3.48, N 2.06; (**N**_**4**_ **CMP-4**) C 95.51, H 3.84, N 0.65; Found C 93.84, H 4.05, N 0.98; (**N**_**4**_ **CMP-5**) C 94.81, H 4.42, N 0.77; Found C 92.84, H 4.75, N 1.04.

## Electronic supplementary material

Controlled synthesis of conjugated polycarbazole polymers via structure tuning for gas storage and separation applications
