# Supplementary material for: Controlled synthesis of conjugated polycarbazole polymers via structure tuning for gas storage and separation applications
Source: Sci Rep. 2017 Nov 13;7:15394. doi: 10.1038/s41598-017-10372-4 (PMC5684200; doi:10.1038/s41598-017-10372-4)
Supplement: Supplementary file 1 — Controlled synthesis of conjugated polycarbazole polymers via structure tuning for gas storage and separation applications [file 41598_2017_10372_MOESM1_ESM.doc]

**Supporting Information**

**Controlled synthesis of conjugated polycarbazole polymers via structure tuning for gas storage and separation applications**

Guoyan Li,*^§^*^a^ Long Qin,*^§^*^a^ Chan Yao^a^ & Yanhong Xu*^a,b^

^a^*K**ey Laboratory of Preparation and Applications of Environmental Friendly Materials of the Ministry of Education, Jilin Normal University, Changchun, 130103, China, Email*: [xuyh@jlnu.edu.cn](mailto:xuyh@jlnu.edu.cn)

^b^*Key Laboratory of Functional Materials Physics and Chemistry of the Ministry of Education, Jilin Normal University, Siping 136000, China*

**Section** **A. Materials and Measurements**

**Section B. The solid-state ^13^CCP-MAS NMR**

**Section** **C. HR-TEM images**

**Section** **D. Electronic absorbance spectra**

**Section** **E. TGA curves**

**Section F. Powder X-ray diffraction patterns**

**Section** **G. Gas adsorption**

**Section** **H. Recyclability for CO_2_ Uptake**

**Section** **I. Gas adsorption at 273 K**

**Section** **J. Reference**

**Section** **A. Materials and Measurements**

Anhydrous DMF, 4,4'-biphenyldiboronic acid, 1,4-diethynylbenzene, 1,3,5-triethynylbenzene, 1,3,6,8-tetrabromocarbazole, tetrakis(triphenylphosphine)palladium (0), and copper (I) iodide were all purchased from Aldrich. 1,1,2,2-Tetrakis(4-ethynylphenyl)ethene and tetrakis(4-bromophenyl)methane were synthesized according to the literature.^S1^ All the solvents used purchased from Aladdin.

^1^H NMR spectra were recorded on Bruker AvanceIII models HD 400NMR spectrometers, where chemical shifts (δ in ppm) were determined with a residual proton of the solvent as standard. Fourier transform Infrared (FT-IR) spectra were recorded on a Perkin-elmer spectrum one model FT-IR-frontier infrared spectrometer. Solid-state ^13^C CP/MAS NMR measurements was recorded using a Bruker AVANCE III 400 WB spectrometer at a MAS rate of 5 kHz and a CP contact time of 2 ms. The solild UV-visible analyzer was used for shimadzu UV-3600. Field-emission scanning electron microscopy (FE-SEM) images were performed on a JEOL model JSM-6700 operating at an accelerating voltage of 5.0 kV. The samples were prepared by drop-casting a THF suspension onto mica substrate and then coated with gold. High-resolution transmission electron microscopy (HR-TEM) images were obtained on a JEOL model JEM-3200 microscopy. Powder X-ray diffraction (PXRD) data were recorded on a Rigaku model RINT Ultima III diffractometer by depositing powder on glass substrate, from 2θ = 1.5° up to 60° with 0.02° increment. The elemental analysis was carried out on a EuroEA-3000. TGA analysis was carried out using a Q5000IR analyser (TA Instruments) with an automated vertical overhead thermobalance. Before measurement, the samples were heated at a rate of 5 °C min^-1^ under a nitrogen atmosphere. Nitrogen sorption isotherms were measured at 77 K with ASIQ (iQ-2) volumetric adsorption analyzer. Before measurement, the samples were degassed in vacuum at 150 °C for 12 h. The Brunauer-Emmett-Teller (BET) method was utilized to calculate the specific surface areas and pore volume. BET surface areas were calculated over the relative pressure range 0.015-0.1 *P*/*P*_0_. Nitrogen NLDFT pore size distributions were calculated from the nitrogen adsorption branch using a cylindrical pore size model. Carbon dioxide, methane and nitrogen sorption isotherms were measured at 298 K or 273 K with a Bel Japan Inc. model BELSORP-max analyzer, respectively. In addition, carbon dioxide sorption isotherms were measured at 318 K and 50 bar with a iSorb HP2 analyzer. Before measurement, the samples were also degassed in vacuum at 120 °C for more than 10 h.

**Section B. The solid-state ^13^CCP-MAS NMR**


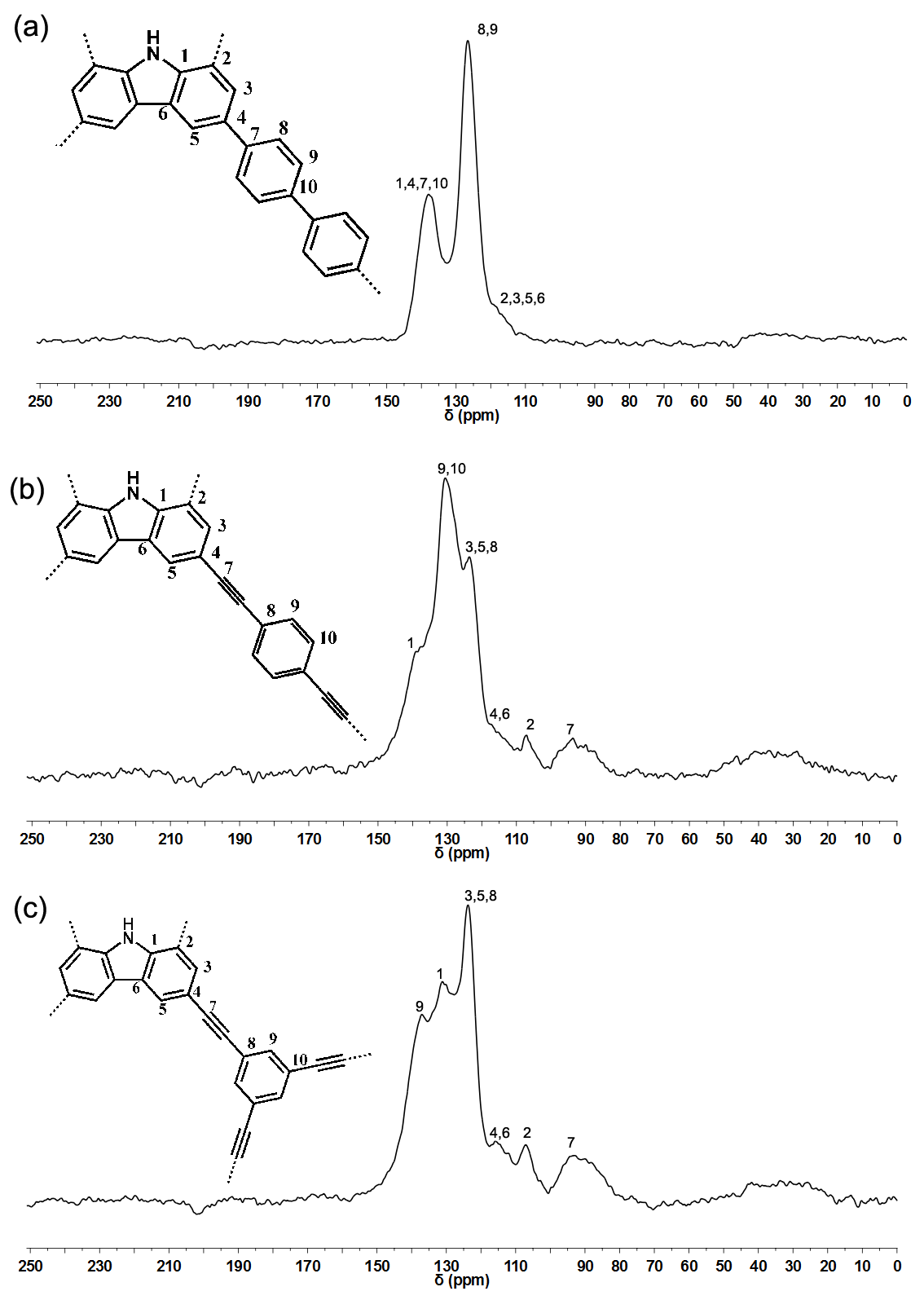


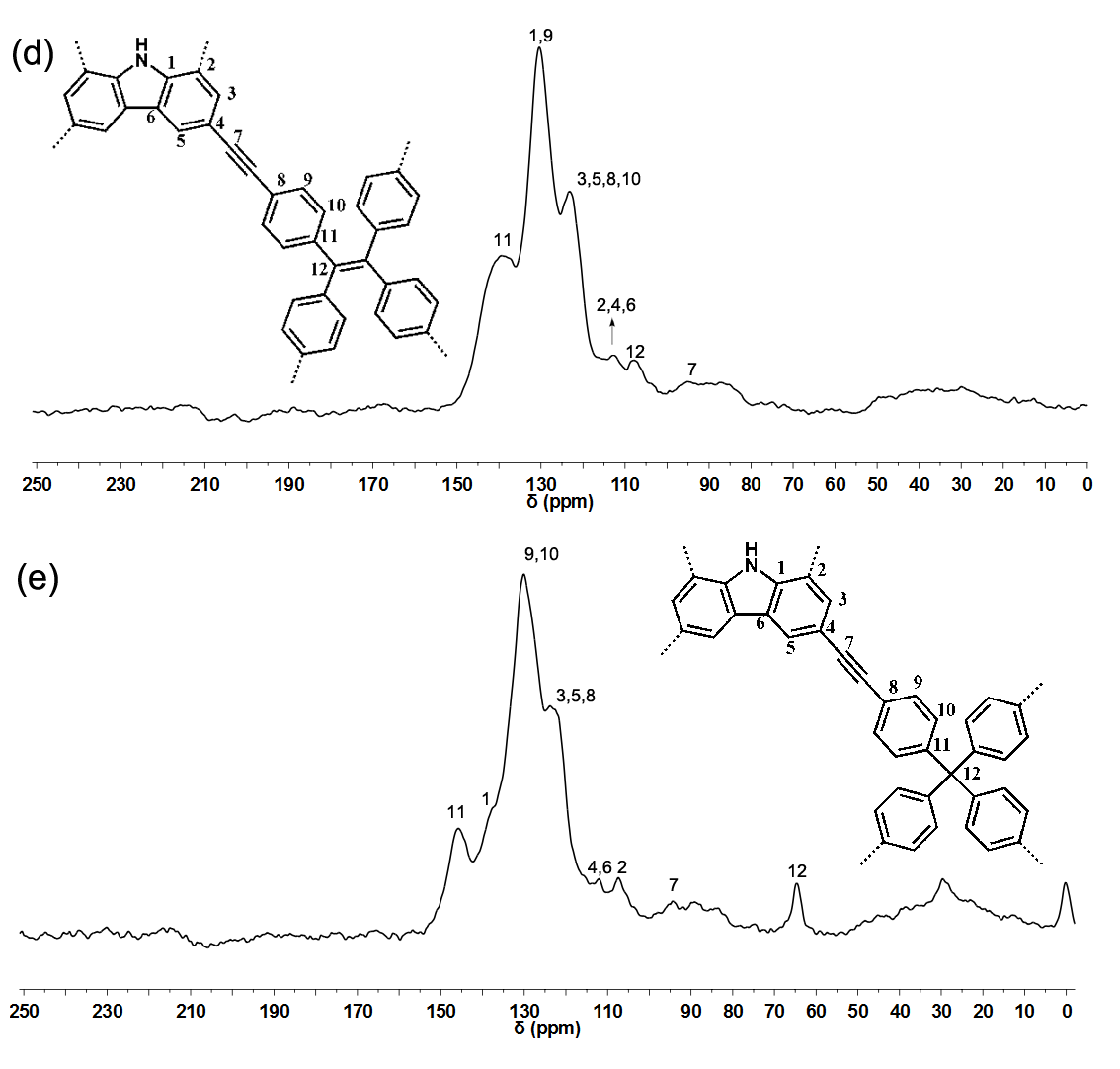


**Figure S1︱** The solid-state ^13^C CP-MAS NMR of (a) N_4_CMP-1, (b) N_4_CMP-2, (c) N_4_CMP-3, (d)

N_4_CMP-4, (e) N_4_CMP-5.

**Section** **C. HR-TEM images**

**
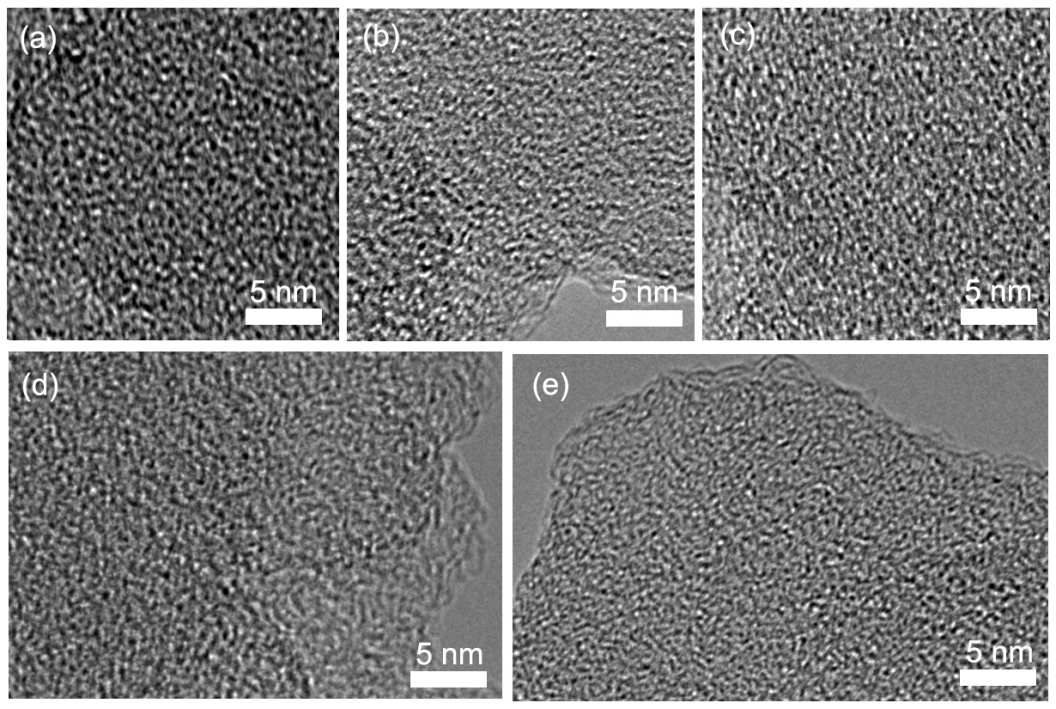
**

**Figure S2︱**HR-TEM images of (a) N_4_CMP-1, (b) N_4_CMP-2, (c) N_4_CMP-3, (d) N_4_CMP-4, and (e) N_4_CMP-5.

**Section** **D. Electronic absorbance spectra**


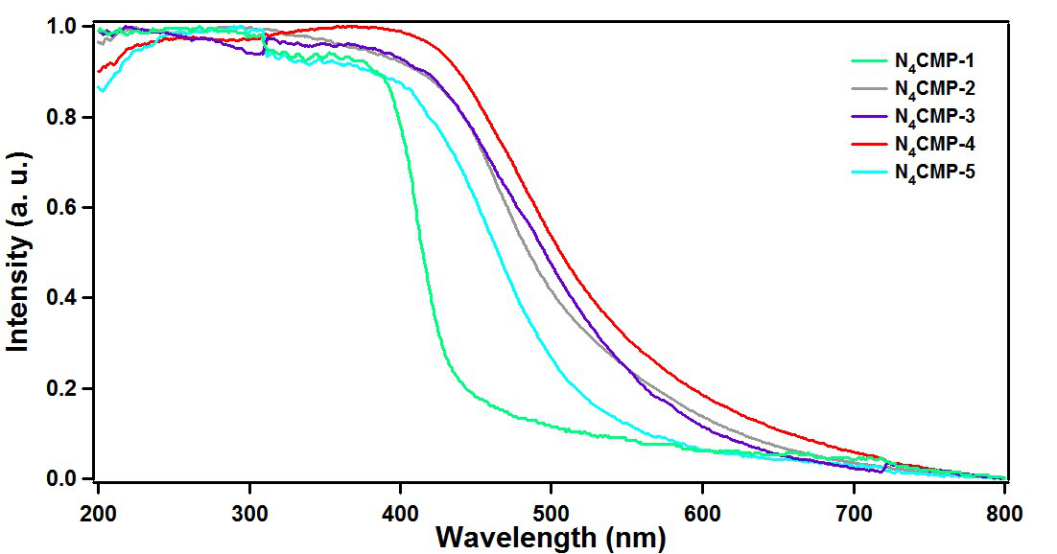


**Figure S3︱**Electronic absorbance spectra of N_4_CMP polymers in solid state.

**Section** **E. TGA curves**


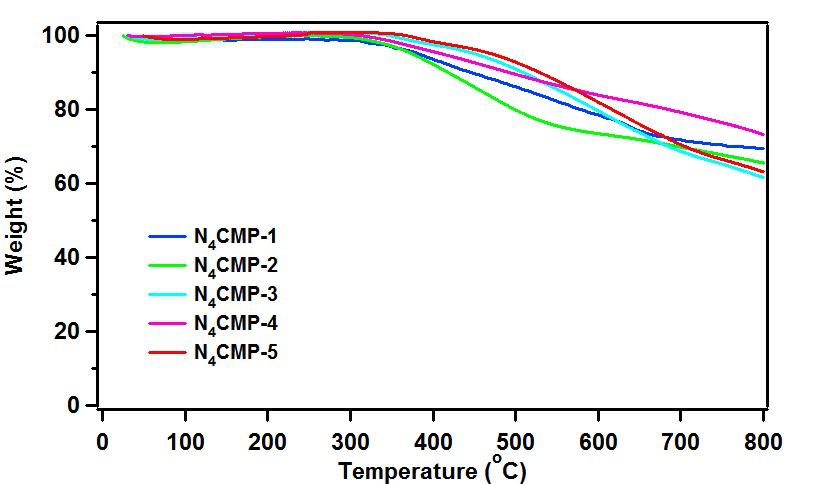


**Figure S4︱**TGA curves of N_4_CMP polymer networks.

**Section** **F. Powder X-ray diffraction patterns**

**
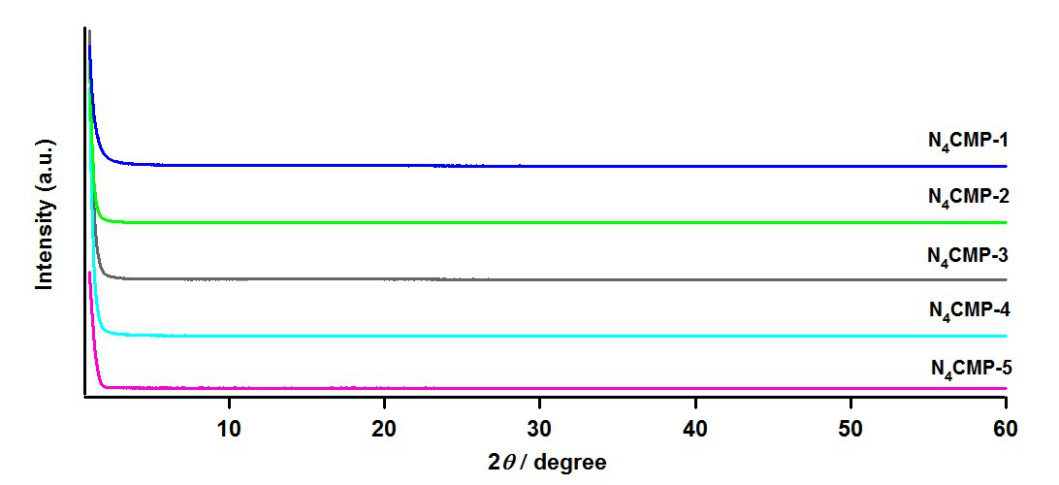
**

**Figure S5︱**Powder X-ray diffraction profiles of N_4_CMP polymer networks.

**Section** **G. Gas adsorption**

**
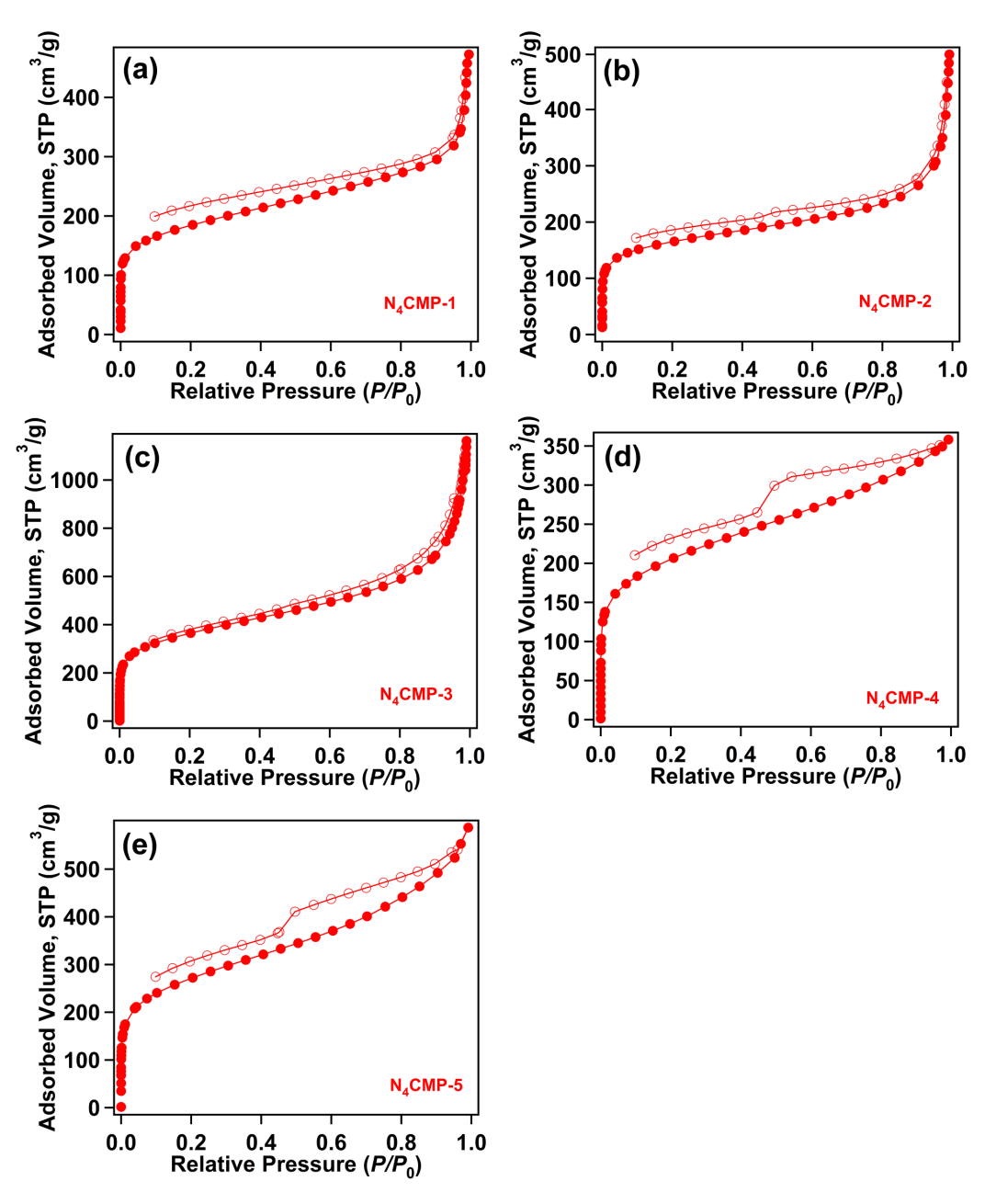
**

**Figure S6︱**Nitrogen sorption isotherms measured at 77 K for N_4_CMP-1–5.


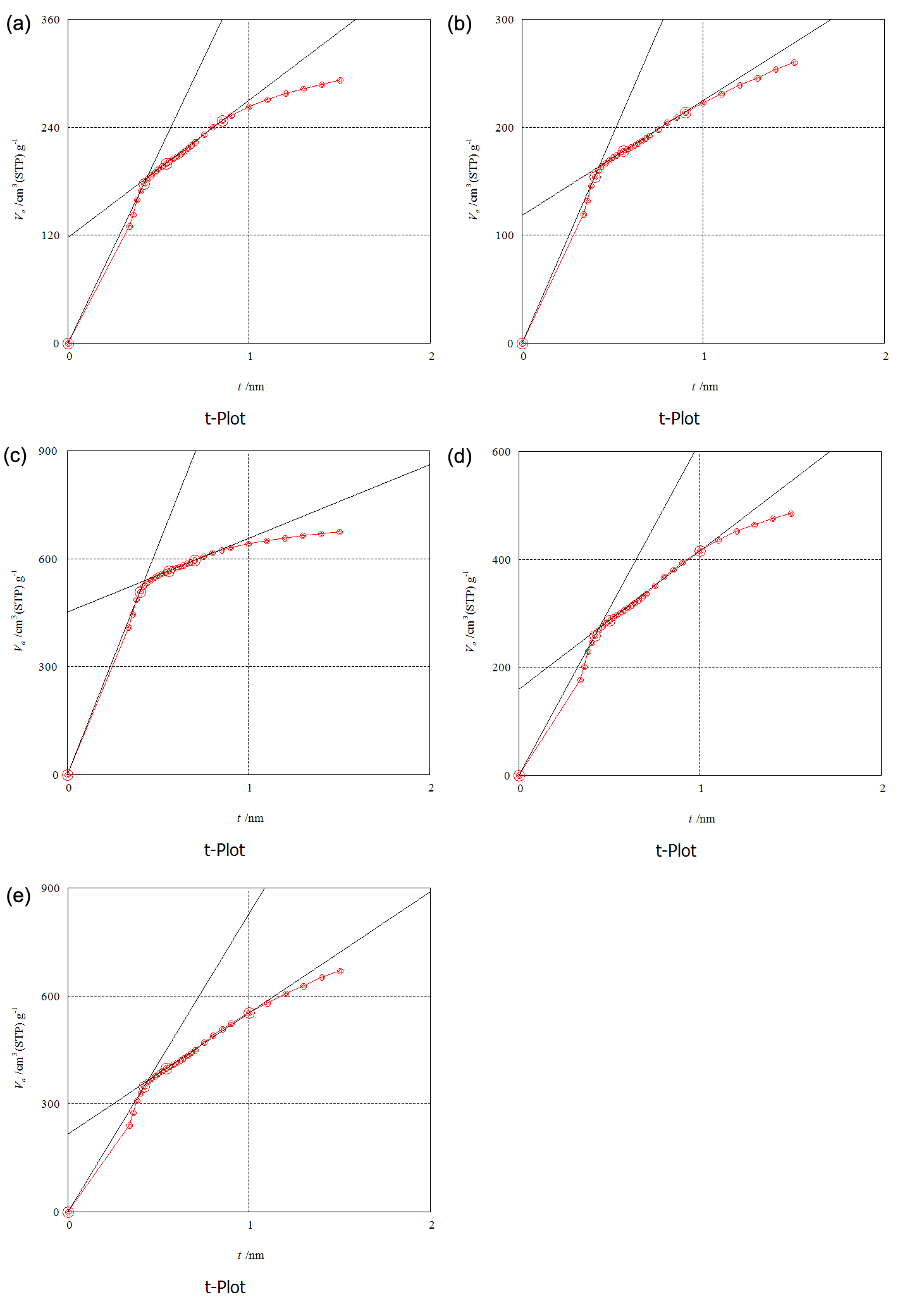


**Figure S7︱**The *t*-plot graphs of (a) N_4_CMP-1, (b) N_4_CMP-2, (c) N_4_CMP-3, (d) N_4_CMP-4, and (e) N_4_CMP-5.


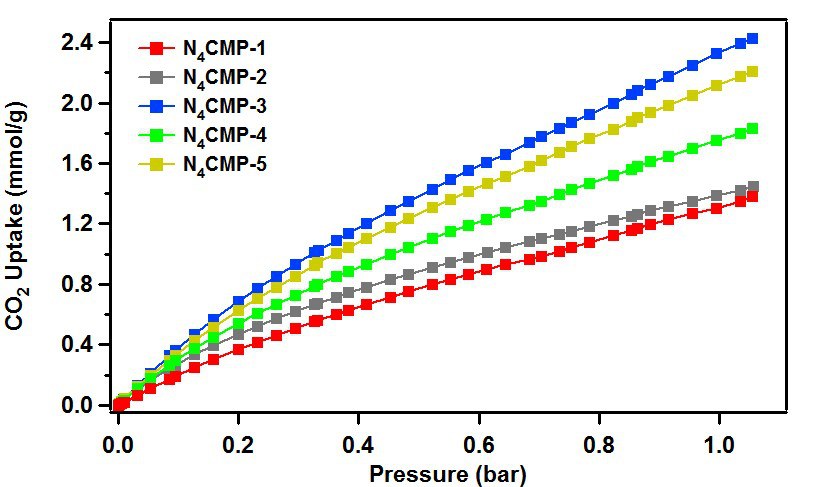


**Figure S8︱**CO_2_ adsorption isotherms collected at 1.05 bar and 298 K.

**Section** **H. Recyclability for CO_2_ Uptake**


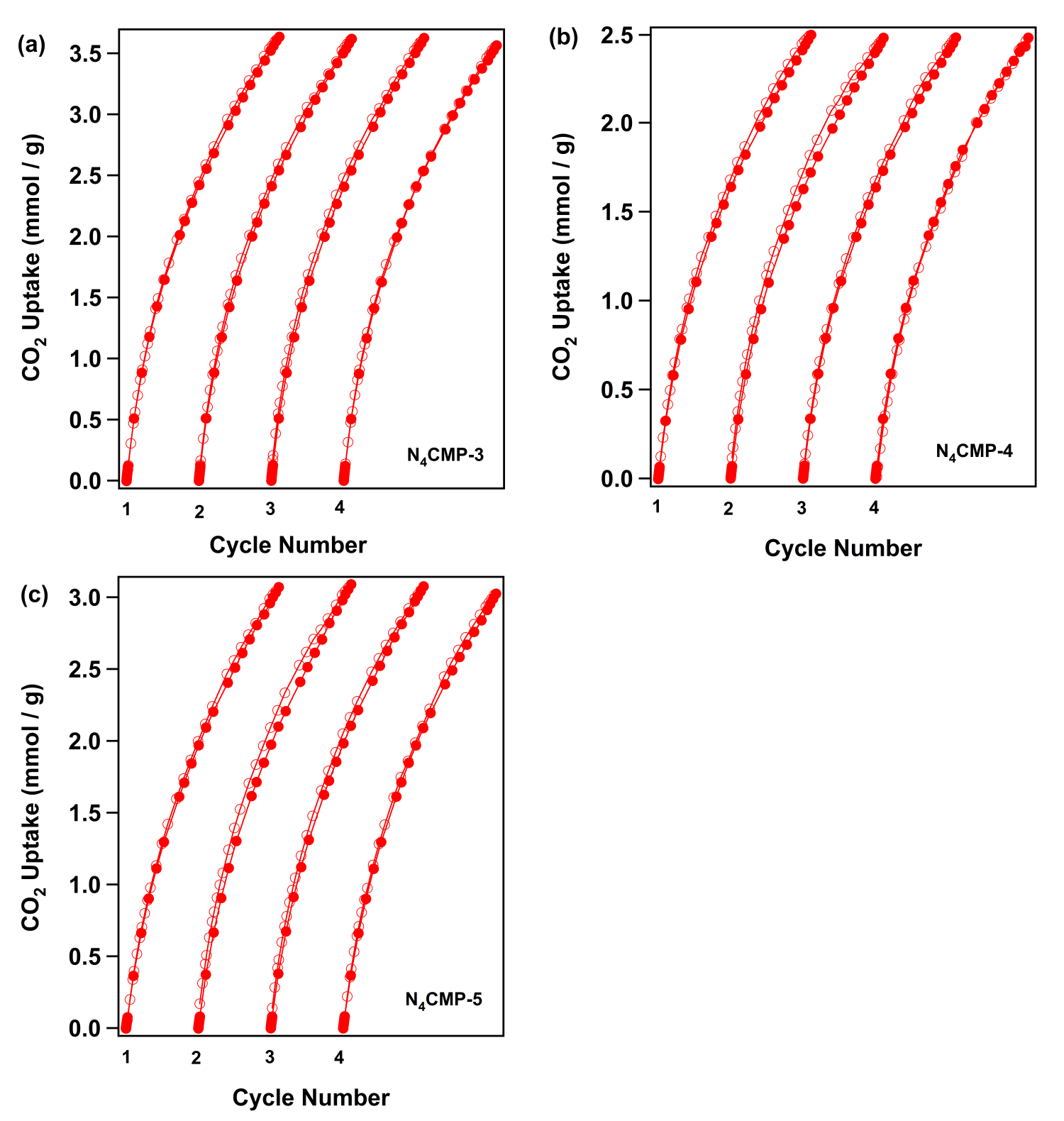


**Figure S9︱**Reusability of N_4_CMP polymers for CO_2_ uptake at 1.05 bar and 273 K.

**Section** **I. Gas adsorption at 273 K**

**
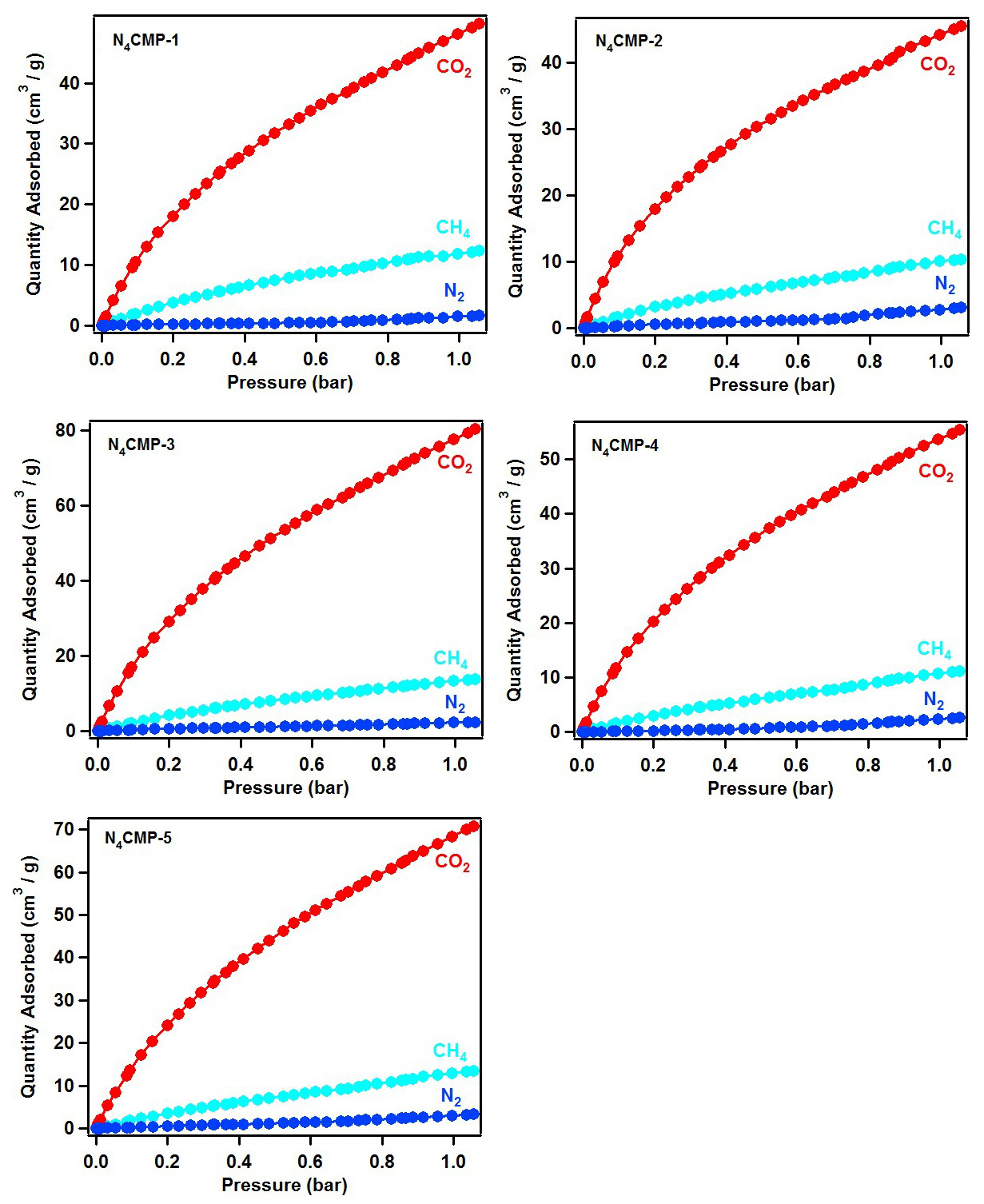
**

**Figure S10︱**Gas adsorption isotherms collected at 1.05 bar and 273 K.


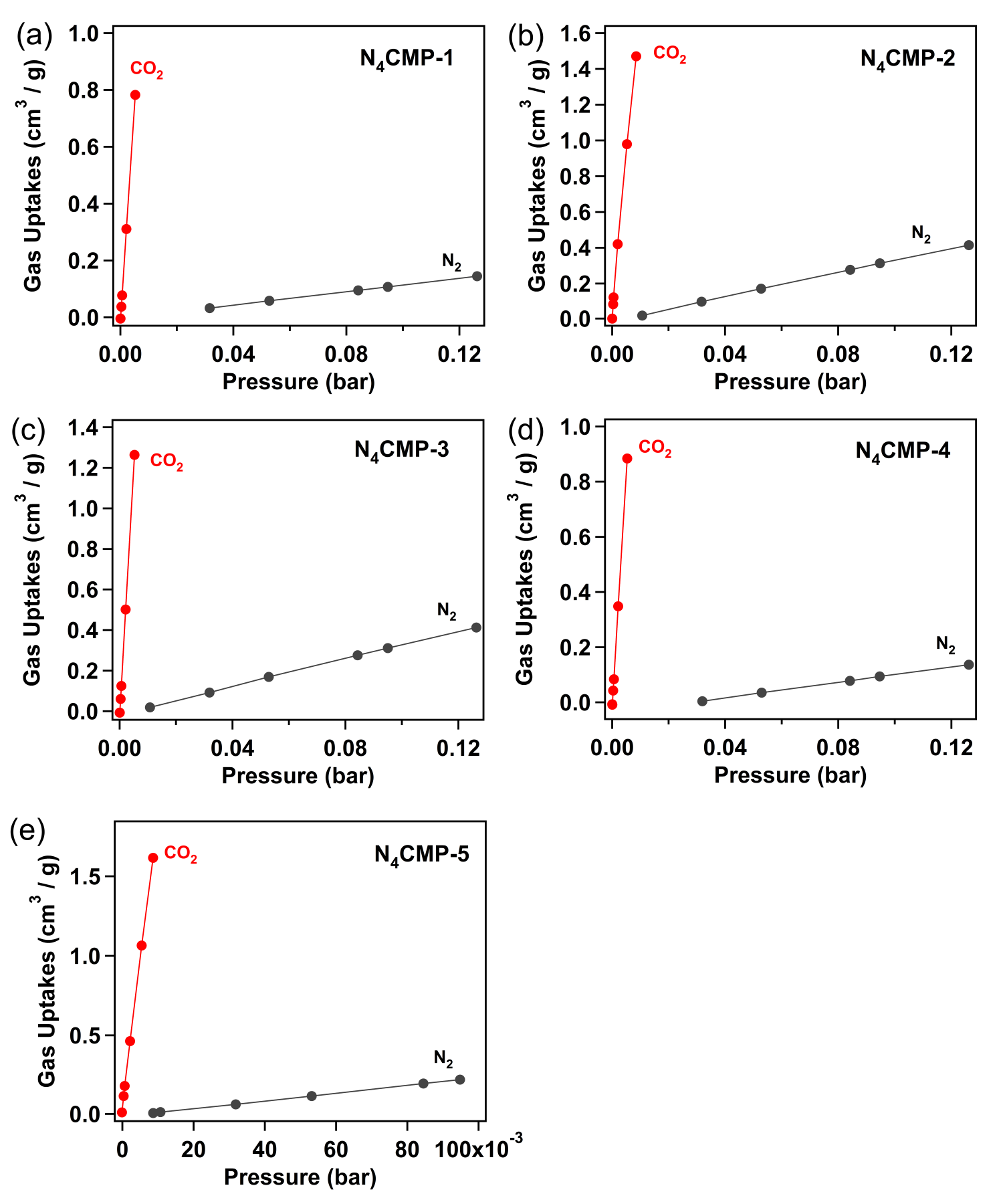


**Figure S11︱**CO_2_/N_2_ initial slop selectivity studies for N_4_CMP-1 (a), N_4_CMP-2 (b), N_4_CMP-3 (c), N_4_CMP-4 (d), and N_4_CMP-5 (e) at 273 K.


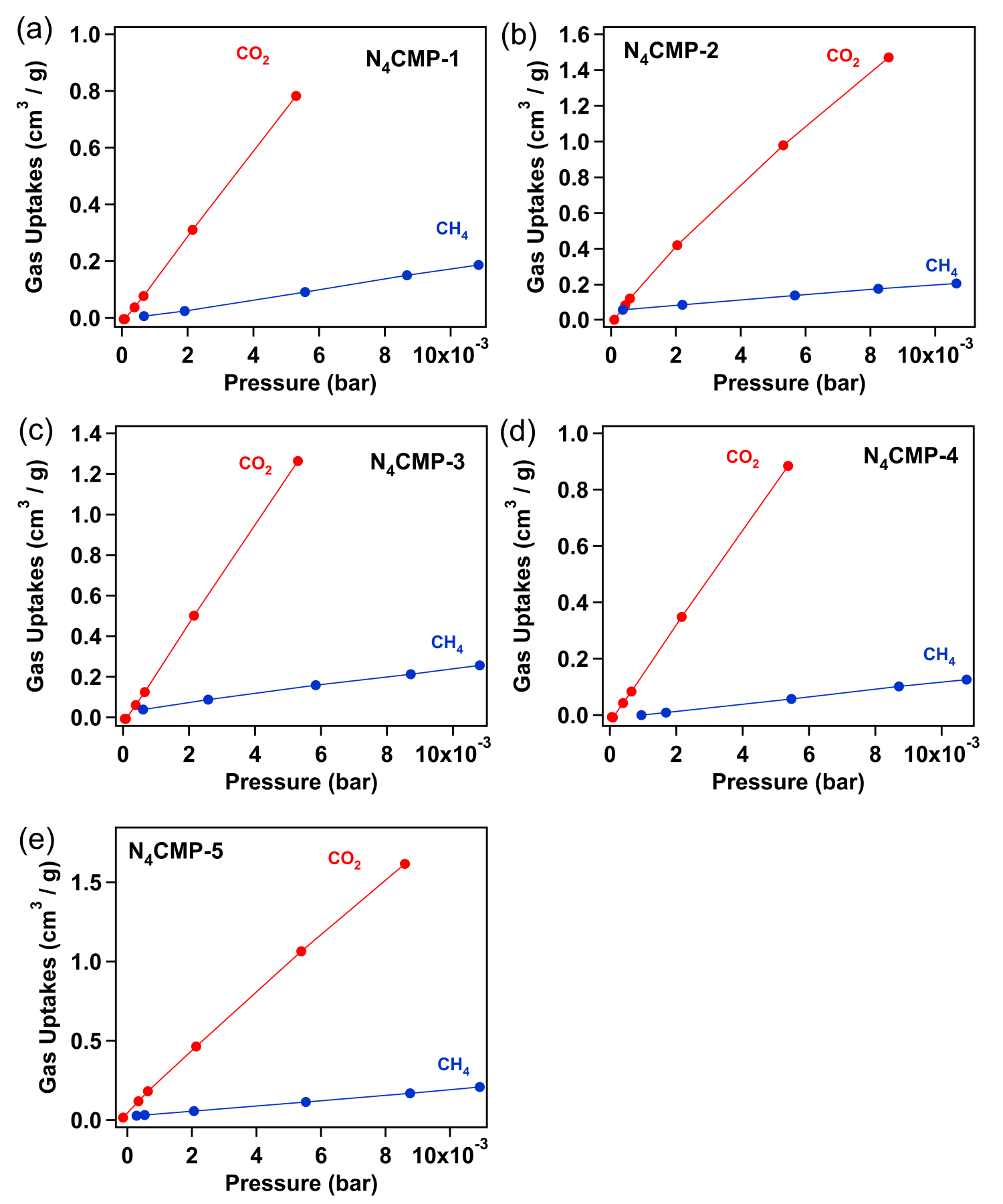


**Figure S12︱**CO_2_/CH_4_ initial slop selectivity studies for N_4_CMP-1 (a), N_4_CMP-2 (b), N_4_CMP-3 (c), N_4_CMP-4 (d), and N_4_CMP-5 (e) at 273 K.


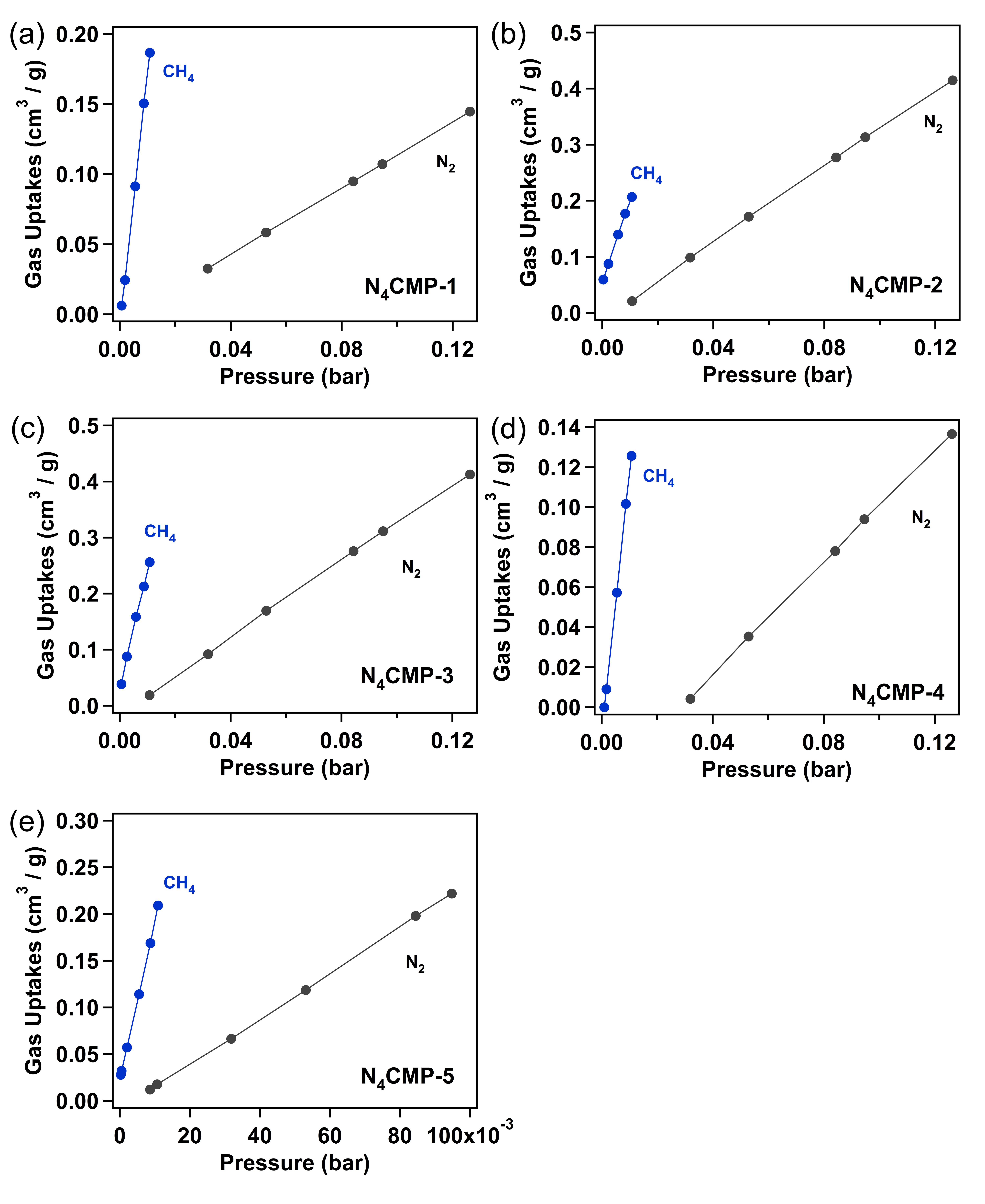


**Figure S13︱**CH_4_/N_2_ initial slop selectivity studies for N_4_CMP-1 (a), N_4_CMP-2 (b), N_4_CMP-3 (c), N_4_CMP-4 (d), and N_4_CMP-5 (e) at 273 K.

**Table S1︱Porosity properties and gas uptake capacities of N_4_CMP.**

| Polymers | *S*_BET_  (m^2^/g)^a)^ | *S*_Micro_  (m^2^/g)^b)^ | *S*_Micro_ / *S*_BET_  (%) | *V*_Total_  (cm^3^/g)^c)^ | *V*_Micro_  (cm^3^/g)^d)^ | *V*_Micro_/  *V*_Total_  (%) | dominant pore size (nm)^e)^ | CO_2_ uptake  (mmol/g)^f)^ | CO_2_/  N_2_  ^g)^ | CO_2_/  CH_4_  ^g)^ | CO_2_/  N_2_  ^h)^ | CO_2_/CH_4_  ^h)^ | CH_4_/N_2_  ^h)^ |
| --- | --- | --- | --- | --- | --- | --- | --- | --- | --- | --- | --- | --- | --- |
| **N_4_CMP-1** | 650 | 430 | 66.1 | 0.712 | 0.569 | 79.9 | 0.51 | 1.38 | 28.7 | 4.6 | 46.2 | 6.4 | 8.1 |
| **N_4_CMP-2** | 592 | 429 | 72.4 | 0.753 | 0.432 | 57.4 | 0.49 | 1.45 | 34.8 | 4.9 | 52.1 | 8.8 | 4.0 |
| **N_4_CMP-3** | 1426 | 758 | 53.1 | 1.775 | 1.287 | 72.5 | 0.36 | 2.43 | 53.8 | 5.2 | 69.1 | 11.4 | 6.1 |
| **N_4_CMP-4** | 995 | 458 | 46.0 | 0.552 | 0.353 | 63.9 | 0.61 | 1.83 | 43.4 | 5.1 | 68.9 | 12.1 | 10.6 |
| **N_4_CMP-5** | 1347 | 643 | 47.7 | 0.906 | 0.667 | 73.6 | 0.56 | 2.21 | 40.2 | 5.0 | 62.5 | 10.8 | 6.7 |

(a) Specific surface area calculated from the adsorption branch of the nitrogen adsorption−desorption isotherm using the BET method; (b) Micropore surface area calculated from the adsorption branch of the nitrogen adsorption−desorption isotherm using the *t*-plot method; (c) Total pore volume at *P*/*P_0_* = 0.99; (d) The micropore volume derived from the *t*-plot method; (e) Data calculated from nitrogen adsorption−desorption isotherms with the NLDFT method; (f) Data were obtained at 1.05 bar and 298 K; (g) Adsorption selectivity based on the IAST method, and (h) Adsorption selectivity based on the Henry’s law.

**Table S2. Summary of CO_2_ sorption properties of porous materials**

| **Adsorbents** | *S*_BET_  (m^2^/g) | CO_2_ uptake (mmol/g)  273 K /1.13 bar | CO_2_ uptake (mmol/g)  298 K /1.13 bar | Selectivity  CO_2_/N_2_  (henry’s law) | Selectivity  CO_2_/CH_4_  (henry’s law) | Ref |
| --- | --- | --- | --- | --- | --- | --- |
| **PPTBC** | 917 | 2.93 | 1.71 | 35.4 | 5.1 | S2 |
| **PMTBC** | 704 | 2.86 | 1.79 | 41.4 | 5.6 | S2 |
| **PPETBC** | 702 | 2.23 | 1.25 | 29.7 | 4.0 | S2 |
| **PMETBC** | 540 | 1.96 | 1.09 | 35.7 | 4.6 | S2 |
| **CPOP-8** | 1610 | 16.5wt% | -^b^ | - | - | S3 |
| **CPOP-9** | 2440 | 18.2wt | - | - | - | S3 |
| **CPOP-10** | 1110 | 14.8wt | - | - | - | S3 |
| **aniline/benzene**  **(0%/100%)^a^** | 1289 | - | 1.61 | 15.9  (IAST) | - | S4 |
| **aniline/benzene**  **(10%/90%)^a^** | 1097 | - | 1.51 | 16.5  (IAST) | - | S4 |
| **aniline/benzene**  **(40%/60%)^a^** | 238 | - | 1.18 | 24.9  (IAST) | - | S4 |
| **aniline/benzene**  **(100%/0%)^a^** | 7 | - | 0.35 | 49.2  (IAST) | - | S4 |
| **CP-CMP1** | 1191 | 3.14 | 1.75 | 21.7 | 3.4 | S5 |
| **CP-CMP2** | 1067 | 3.06 | 1.69 | 22.1 | 4.0 | S5 |
| **CP-CMP3** | 883 | 2.35 | 1.53 | 25.6 | 3.9 | S5 |
| **CP-CMP4** | 1083 | 3.12 | 1.81 | 24.2 | 4.0 | S5 |
| **CP-CMP5** | 2241 | 4.57 | 2.44 | 20.5 | 3.9 | S5 |
| **CP-CMP6** | 1764 | 4.35 | 2.35 | 23.9 | 4.2 | S5 |
| **CP-CMP7** | 847 | 2.80 | 1.66 | 40.6 | 6.0 | S5 |
| **P-PCz^b^** | 1647 | 5.57 (1.0 bar) | 2.97 | 32 | - | S6 |
| **N_4_CMP-3** | 1426 | 3.62  (1.05 bar) | 2.43  (1.05 bar) | 53.8  (IAST) | 5.2  (IAST) | This work |

^a^CO_2_ uptake of polymers were obtained at 300 K and 1 bar, ^b^ Not mentioned

**Section** **J. Reference**

S1 (a) Wang, J., *et al*. Ethynyl-capped hyperbranched conjugated polytriazole: click polymerization, clickable modification, and aggregation-enhanced emission. *Macromolecules* **45**, 7692-7703 (2012); (b) Li, P. Z., *et al*. A triazole-containing metal–organic framework as a highly effective and substrate size-dependent catalyst for CO_2_ conversion. *J. Am. Chem. Soc.* **138**, 2142-2145 (2016).

S2 Wang, X., *et al*. [Synthetic control of pore properties in conjugated microporous polymers based on carbazole building blocks.](http://onlinelibrary.wiley.com/doi/10.1002/macp.201400508/full) *Macromol. Chem. Phys.* **216**, 504-510 (2015).

S3 Chen, Q., Liu, D., Zhu, J. & Han, B. Mesoporous conjugated polycarbazole with high porosity via structure tuning. *Macromolecules* **47**, 5926-5931 (2014).

S4 Dawson, R., *et al*. [Microporous copolymers for increased gas selectivity](http://pubs.rsc.org/en/content/articlelanding/2012/py/c2py20136d). *Polym. Chem.* **3**, 2034-2038 (2012).

## S5 Yu, M., *et al*. Conjugated microporous copolymer networks with enhanced gas adsorption.

*Polym. Chem.* **6**, 3217-3223 (2015).

S6 Jin, T., *et al*. Rational design and synthesis of a porous, task-specific polycarbazole for efficient CO_2_ capture. [*Chem. Commun.*](http://pubs.rsc.org/en/journals/journal/cc) **52**, 4454-4457 (2016).
